# Domperidone Prescribing Practices Exposed Patients to Cardiac Risk despite a “Black Box” Warning: A Canadian Tertiary Care Center Study

**DOI:** 10.1155/2016/2937678

**Published:** 2016-02-24

**Authors:** Nauzer Forbes, Mohan Cooray, Raed Al-Dabbagh, Yuhong Yuan, Frances Tse, Louis W. C. Liu, Ted Xenodemetropoulos

**Affiliations:** ^1^Division of Gastroenterology, McMaster University Department of Medicine, Hamilton, ON, Canada L8S 4K1; ^2^Division of Gastroenterology, University of Calgary Therapeutic Endoscopy Training Program, Calgary, AB, Canada T2N 4Z6; ^3^Farncombe Family Digestive Research Institute, Hamilton, ON, Canada L8S 4K1; ^4^Division of Gastroenterology, University of Toronto Department of Medicine, Toronto, ON, Canada M5T 2S8

## Abstract

*Background.* In 2012, Health Canada released a warning regarding domperidone use, based on associations with life-threatening arrhythmias and death.* Objective.* This study aimed to compare the appropriateness of domperidone prescribing patterns before the advisory to those afterward.* Methods.* Two retrospective reviews were conducted for patients prescribed domperidone during quarters in 2005 and 2012. Outcomes included appropriateness of indication, dosing regimens, monitoring of electrolytes, baseline electrocardiogram performance and characteristics, presence of left ventricular dysfunction, and coprescription of QT-prolonging medications. Univariable and multivariable logistic regression analyses were performed. *p* values < 0.05 were considered significant.* Results.* 290 and 287 patients were analyzed in 2005 and 2012, respectively. Domperidone initiation in hospital decreased from 2005 to 2012 (71.4% versus 39.4%, *p* < 0.0001) as did prescriptions for nonapproved indications (84.8% versus 58.2%, *p* < 0.0001). In-hospital initiation predicted prescription for nonapproved indications (OR = 7.01, 95% CI 4.52–10.87, *p* < 0.0001). Use of domperidone as the sole GI drug predicted nonapproved indications (OR = 2.51, 95% CI 1.38–4.55, *p* = 0.002).* Conclusions.* The advisory was associated with more appropriate domperidone initiation and compliance with recommended dosages. Our study suggests the need for increased awareness of the dosing and monitoring of domperidone to ensure patient safety.

## 1. Introduction

Domperidone, a benzimidazole-derived dopamine receptor antagonist, exhibits both prokinetic and antiemetic properties. Domperidone's higher molecular weight and lower lipophilicity are felt to contribute to its relative inability to cross the blood brain barrier and, thus, lesser incidence of central adverse effects in comparison to metoclopramide. Current Health Canada approved indications for domperidone use are symptomatic management of upper gastrointestinal motility disorders associated with chronic and subacute gastritis, diabetic gastroparesis, and prevention of gastrointestinal symptoms associated with the use of dopamine agonist anti-Parkinsonian agents.

In March of 2012, Health Canada issued an alert [1] regarding domperidone use, warning practitioners about the risk of ventricular tachyarrhythmias and sudden cardiac death with its use, particularly in doses exceeding 30 mg/day, and in patients older than 60. This information was based primarily on two studies: a population-based case-control study [2] that showed increased risk of sudden cardiac death, which was higher in patients using daily doses >30 mg, with an adjusted OR = 11.4 (1.99–65.2), and a nested case-control study [3] that showed an increased risk of a composite endpoint of sudden cardiac death and serious ventricular arrhythmia, which was higher in patients older than 60 years of age (adjusted OR 1.64, 1.31–2.05).

Numerous unapproved or “off-label” clinical uses of domperidone exist, including gastroesophageal reflux disease (GERD), the induction and maintenance of adequate lactation in breast-feeding women [4], treatment of vague dyspepsia symptoms, and treatment of nausea and/or emesis, especially in the postoperative or postchemotherapy settings. In the hospital environment, the drug is frequently used in patients fed via nasogastric tube to promote kinesis and clear residual volumes. In spite of the potentially lethal consequences of its use, data on the prescription practices of domperidone by Canadian physicians are lacking in the literature and thus need to be defined.

The objectives of this study were threefold. The first was to review the appropriateness of domperidone prescription in relation to the approved indications in admitted patients at 2 tertiary care hospitals within a single Canadian city. The second was to compare domperidone prescribing patterns before the 2012 Health Canada advisory to those afterward, thereby assessing its impact. The final aim was to determine physician compliance with regard to measuring baseline safety parameters in patients prescribed domperidone.

## 2. Methods

### 2.1. Study Setting and Design

Hamilton Health Sciences (HHS) is a tertiary care hospital network serving over 2.3 million residents of Hamilton and south central Ontario and is the second largest hospital group in Ontario. HHS encompasses over 1100 inpatient beds; this includes patients admitted to medicine, surgery, and critical care services, all of which were considered for this study. Two retrospective electronic medical record reviews were conducted for patients at 3 centers (McMaster University Medical Centre, Hamilton General Hospital, and Juravinski Hospital) within the HHS network. The reviews were conducted for consecutive hospital inpatients who received prescriptions for domperidone over 4-month periods (April–July) in 2005 and 2012, respectively. Ethics approval for the study was obtained through the Hamilton Integrated Research Ethics Board at McMaster University.

### 2.2. Study Patients

Study patients were compiled from the centralized HHS pharmacy computer database and included those initially prescribed domperidone during their hospital admission as well as those with preexisting prescription prior to their hospital admission. Any patient aged 18 and over who met the above criteria was included. Furthermore, the dose and duration of domperidone prescription were also recorded.

### 2.3. Outcomes

Consistency of domperidone indication with approved indications was assessed, based on current Health Canada recommendations. The medical records of all study patients were reviewed for indications of domperidone prescription that were either explicitly documented or inferred in the medical history. In addition, dosing regimens were assessed, including any observed changes to dosages. Outcome measures assessed were derived from the most recent Health Canada document on Guidance for Product Monograph Content for QT/QTc Interval Prolongation [5]. This included whether or not an electrocardiogram was performed during the patient's admission, as well as whether there was any evidence of corrected QT interval (QTc) prolongation, defined as a period equal to or greater than 440 msec. Further outcome measures included the monitoring of serum potassium (K^+^), magnesium (Mg^+^), and calcium (Ca^2+^) levels, either during the first 2 days of initial domperidone prescription or on admission to hospital for patients already on domperidone therapy prior to their admission. Finally, the presence of left ventricular (LV) dysfunction by transthoracic echocardiogram and coprescription of other known QT-prolonging medications were also assessed. Potential QT-prolonging medications were cross-referenced and confirmed using updated drug databases [6].

### 2.4. Data Analysis

Data was analyzed using the Statistical Package for the Social Sciences (SPSS) software, version 20. *χ* ^2^ test was used to assess significance of differences in proportions. A *p* value of <0.05 was considered statistically significant. An independent *t*-test was used to determine if there was a difference in mean age between groups. Differences in outcomes between 2005 and 2012 were evaluated by univariable analyses. Potential predictors for nonapproved indications were preselected and then analyzed by multivariable logistic regression. These predictors included age, daily domperidone dosage, and timing of initial prescription (i.e., prior to or during hospitalization). Within patients who were initially prescribed domperidone during their hospital admission, the degree of inappropriate initiation of their medication was measured by multivariable logistic regression analyses and was compared between 2005 and 2012.

## 3. Results

A total of 577 patients, aged 19 to 98, over two study periods were identified, and their medical records were reviewed. The two 4-month periods were April to July in 2005 and April to July in 2012. The second period occurred soon after the release of the Health Canada advisory in March of 2012 [1]. The baseline characteristics of patients and their respective domperidone prescriptions are shown in Table 1. After the advisory in 2012 (compared to 2005), significantly less domperidone was initially prescribed in hospital, and significantly more prior domperidone prescriptions were discontinued during hospitalization (Table 1). Furthermore, after the advisory, significantly less domperidone was prescribed at potentially hazardous doses, and there were significantly fewer prescriptions for unclear or nonapproved indications (Table 1). Unclear or nonapproved use of domperidone was recorded based on lack of documentation of indication(s) in the medical record after close review of the details of the admission and the past medical history.

A multivariable regression model of preselected variables revealed that in-hospital initiation highly predicted prescription for nonapproved indications (OR = 7.01, 95% CI 4.52–10.87, *p* < 0.0001). In addition, the use of domperidone as the sole GI drug also significantly predicted prescription for nonapproved indications (OR = 2.51, 95% CI 1.38–4.55, *p* = 0.002). Patient age did not significantly predict prescription for nonapproved indications (mean age = 68.2 for approved indications, mean age = 63.9 for nonapproved indications, *p* = 0.23). Finally, a cumulative domperidone dose of greater than 30 mg/day did not significantly predict prescriptions for nonapproved indications (OR = 1.18, 95% CI 0.75–1.85, *p* = 0.47).

Multivariable regression was also performed to assess differences in patient safety and monitoring parameters between the two study periods for patients who were initially prescribed domperidone in hospital. Initiation of domperidone was considered to be “inappropriate”: if an electrocardiogram (ECG) was not performed prior to the start of the medication, if QTc interval was prolonged (>440 msec), if electrolyte levels (Ca^2+^, K^+^, Mg^2+^) were unmeasured or abnormal, if left ventricular (LV) function was depressed with ejection fraction (EF) < 40%, or if there was concurrent use of other QT-prolonging drugs. Differences in the inappropriate initiation of domperidone between 2005 and 2012 are presented in Table 2.

## 4. Discussion

This retrospective study in a single tertiary care center has shown more appropriate use of domperidone following the 2012 Health Canada advisory. However inappropriate or unclear utilization of domperidone continues to be high, occurring in 72% of the studied patients. Predictors of nonapproved indications included in-hospital initiation of the drug, as well as use of domperidone as a sole GI agent. The concurrent use of other QT-prolonging medications during domperidone prescription was also common. Several key differences in prescribing patterns were observed before and immediately after the Health Canada warning in 2012 [1]: significantly less domperidone was initiated in hospital (71.4% versus 39.4%, *p* < 0.0001), significantly less high dose domperidone was prescribed for patients aged greater than 60 (40.7% versus 28.9%, *p* = 0.01), and significantly less domperidone was prescribed for nonapproved indications (84.8% versus 58.2%, *p* < 0.0001). Furthermore, baseline metabolic and cardiac risk assessment was not routinely performed in association with domperidone prescription, although there was significant improvement in 2012 compared to 2005.

QT interval prolongation has been used as a surrogate marker for predicting adverse and potentially lethal medication-induced effect related to torsades de pointes, both clinically and in research settings [7]. Domperidone has been frequently associated with both QT interval prolongation and ventricular tachyarrhythmia in the literature, with cardiotoxicity being described with both oral and intravenous administration [2, 3, 8–10]. Domperidone's risk of sudden cardiac death may be comparable to cisapride, a prokinetic medication that has long been removed from the North American pharmaceutical market following postmarketing surveillance reports of elevated risk of QT interval prolongation and torsades de pointes, as well as arrhythmia-related fatality [11–13]. Therefore, dissemination of these concerns and resultant uptake of safer prescribing practices are crucial.

### 4.1. Strengths and Limitations

Our study design took advantage of the timing of the March 2012 Health Canada advisory in order to compare tertiary care hospital-based prescribing practices before and after the warning. Although our study could not establish causality of the change in prescribing pattern of domperidone as a consequence of the dissemination of the 2012 Health Canada warning, it clearly demonstrated the increased awareness of its appropriate use. Further studies are required to confirm its impact on prescribing practices in outpatient clinics and community hospitals.

Many inherent limitations apply to our retrospective chart-review study. A proportion of the studied patients had no clearly documented or inferable indication for domperidone prescription on available hospital records. There was also a subset of diabetic patients on domperidone with no clearly documented history of diabetic gastroparesis or objective evidence of delayed gastric emptying. Although these patients may indeed reflect the general trend of prescription of domperidone for unapproved therapeutic uses, it is possible that improper documentation overestimated the proportion of nonapproved indications in this study. Similarly, there was an inability to assess whether the electrolyte measurements and ECGs performed during a given patient's hospital admission were directly related to the initiation of domperidone. Coincidental performance of these tests for other medical assessments could not be determined, which may overestimate the degree of safety with which domperidone was prescribed. Furthermore, our study was unable to establish causality between domperidone prescription and adverse outcomes such as cardiac arrhythmia or increased mortality due to the retrospective design and relative sample sizes.

## 5. Conclusion

Our study demonstrates that heightened awareness of the appropriate use and monitoring of domperidone are required. The 2012 Health Canada advisory warning significantly improved the use of domperidone in terms of appropriate indications, dosing, and monitoring. Further qualitative research employing a physician survey-based design could be useful in establishing the causality between the Health Canada advisory and changes in practice patterns. Increased in-hospital awareness of domperidone's indications and adverse effects, through both active and passive techniques, could serve to enhance the impact of the Health Canada advisory. Increased awareness of the indications, dosage, and adverse effects of domperidone will result in improved patient safety, quality of care, and healthcare expenditure.

## Figures and Tables

**Table 1:** Baseline domperidone prescribing practices in 2005 and 2012 (after Health Canada advisory).

	2005	2012	*p* value
	*n* = 290	*n* = 287	
Characteristic			
Mean age	62.4	67.9	<0.001
Initially prescribed in hospital	207 (71.4%)	113 (39.4%)	<0.001^*∗*^
Prior prescription stopped in hospital	1 (0.3%)	19 (6.6%)	<0.001^*∗*^
Dose > 30 mg/day	190 (65.5%)	136 (47.4%)	<0.001^*∗*^
Dose > 30 mg/day in patients > 60 y.o.	118 (40.7%)	83 (28.9%)	0.01^*∗*^
Indications			
GERD	33 (11.4%)	86 (30.0%)	<0.001^*∗*^
Gastroparesis	8 (2.8%)	24 (8.4%)	0.003^*∗*^
Dyspepsia	3 (1.0%)	1 (0.3%)	0.6
Antiemesis	12 (4.1%)	7 (8.4%)	0.04^*∗*^
Parkinson's with anti-Parkinsonian drugs	1 (0.3%)	3 (1.0%)	0.4
Unclear or nonapproved indications	246 (84.8%)	167 (58.2%)	<0.001^*∗*^

Note: GERD = gastroesophageal reflux disease.

^*∗*^Significant difference in domperidone prescribing patterns between 2005 and 2012 (*p* value < 0.05 by *χ* ^2^ test of difference).

**Table 2:** Inappropriate assessment prior to inpatient domperidone initiation in 2005 and 2012 (after Health Canada advisory).

	2005	2012	OR (95% CI)
	*n* = 207	*n* = 113	
Safety/monitoring parameter			
ECG not performed prior to initiation	77 (37.2%)	19 (16.8%)	0.032 (0.006–0.156)^*∗*^
QTc interval prolonged (>440 msec)	170 (82.1%)	76 (67.3%)	0.025 (0.005–0.119)^*∗*^
Ca^2+^ level unmeasured or abnormal	117 (56.5%)	32 (28.3%)	0.311 (0.147–0.651)^*∗*^
K^+^ level unmeasured or abnormal	77 (37.2%)	24 (21.2%)	0.476 (0.204–1.109)
Mg^2+^ level unmeasured or abnormal	42 (20.3%)	29 (25.7%)	1.623 (0.726–3.625)
LV function depressed (EF < 40%)	99 (47.8%)	11 (9.7%)	0.220 (0.101–0.481)^*∗*^
Concurrent use of other QT-prolonging drug(s)	123 (59.4%)	55 (48.7%)	0.744 (0.375–1.476)

Note: OR = odds ratio, CI = confidence interval, ECG = electrocardiogram, Ca^2+^ = serum calcium, K^+^ = serum potassium, Mg^2+^ = serum magnesium, LV = left ventricular, and EF = ejection fraction.

^*∗*^Significant difference in domperidone prescribing patterns between 2005 and 2012.
